# Abatement effects of different soil amendments on continuous cropping of *Codonopsis pilosula*

**DOI:** 10.3389/fpls.2024.1376362

**Published:** 2024-04-19

**Authors:** Zhaodi Yang, Daiyu Qiu, Kan Jiang, Maoxiao Du, Hongyan Li

**Affiliations:** ^1^ State Key Laboratory of Crop Stress Biology for Arid Areas, Agricultural College of Gansu Agricultural University, Lanzhou, China; ^2^ Herbal Resources Department, Guangdong Yifang Pharmaceutical Co., Ltd, Foshan, China

**Keywords:** *Codonopsis pilosula*, continuous cropping obstacle, biochar, bacterial fertilizer, vermicompost

## Abstract

**Introduction:**

*Codonopsis pilosula* is widely sought-after in China as a substitute for the more expensive ginseng. Continuous cropping of *C. pilosula* supports a vibrant health-supplement industry but requires significant inputs of fertilizers which increase production costs and degrade the environment.

**Methods:**

Here, three environmentally-friendly natural fertilizers, including biochar, bacterial fertilizer, and vermicompost, were used at different concentrations (undiluted, diluted 10 times, diluted 50 times) to determine their efficacy in seed germination and growth physiology of *C. pilosula* in continuous cropping.

**Results:**

The results showed that biochar, bacterial fertilizer, and vermicompost with different concentrations of leachate could all increase the germination rate, germination potential and germination index of *C. pilosula* seeds treated with inter-root soil leachate of continuous *C. pilosula*; increase the activity of antioxidant enzymes (superoxide dismutase and peroxidase) in *C. pilosula* seedlings under the stress of inter-root soil leachate of continuous *C. pilosula*, reduce the over-accumulation of malondialdehyde (MDA) content, and increase the resistance of *C. pilosula* seedlings. After transplanting, superoxide dismutase (SOD) activity increased by an average of 16.1%. Peroxidase (POD) levels showed an average increase of 16.4%. Additionally, there was a significant reduction in the MDA content, with an average decrease of 50%, and the content of osmotic-regulating substances (free proline content and soluble protein content) exhibited a significant increase.

**Discussion:**

In conclusion, biochar, bacterial manure, and vermicompost have the potential to overcome the challenges of extensive fertilizer use in continuous cropping of *C. pilosula*.

## 1 Introduction

The Chinese herbal medicine *Codonopsis pilosula* has been recognized for its ability to strengthen the spleen and lungs, nourish the blood, generate fluids, and supplement human diet. It has been listed in the catalog of medicinal food, and its market demand has shown a rising trend in 2018 (Huang et al., 2021). Gansu Province in China is the main production area of *C. pilosula*, and the main cultivated species is *Codonopsis pilosula Nannf.* var. *modesta* (Nannf.) L.T. Shen (National Pharmacopoeia Committee, 2020).

Obstacles to continuous cropping are defined as the abnormal growth and development of crops caused by continuous cultivation of the same crop or related crops in the same field. Currently, China’s grain crops, oilseed crops, vegetables, melons, fruits, and other cash crops, along with a majority of medicinal plants, are grown in a continuous cropping system which often fails various degrees of continuous cropping obstacles due to poor quality of soil, soil degradation, and nutrient depletion (Liu et al., 2010; Durre et al., 2023). As a result, there has been a significant decline in both yield and quality of crops. Continuous cropping obstacle can significantly reduce the yield of *Asparagus officinalis* and a 15% reduction in yield and deterioration in the quality of rice (Shi et al., 2018; Yin et al., 2019). Grapes grown in a continuous cropping system exhibit decreased plant growth and higher incidence of disease (Shi et al., 2018; Yin et al., 2019).

Medicinal plants may be more affected than vegetable and fruit crops. Studies indicate that affects more than 70% of plant roots, and this limits the growth of *Panax quinquefolius*, *Panax notoginseng*, and *Radix ginseng* as succession plants. After nearly a year of continuous *Radix ginseng* and *Panax quinquefolius* growth, seedling survival plummeted to 30%. *Panax notoginseng* perished in severe circumstances of continuous cropping, due to its tractability and specificity of growth. In general, *Panax notoginseng* can only be replanted every 10 years (Guo et al., 2006; Jian et al., 2008; Zhao et al., 2014).

Chemosensitizers, which cause the continuous cropping obstacle to develop, are typically distributed in plant reproductive and some nutrient organs. They can be released into the environment via a number of different pathways, including exudation with root secretions (Deng et al., 2017; Jouni et al., 2023; Nwe et al., 2023). The germination and seedling growth may be hindered by autotoxic chemicals found in soil extracts (He et al., 2018). In a study by Zhao et al. (Zhao et al., 2023), the root secretions of *Salvia miltiorrhiza* under continuous cropping exhibited inhibitory and toxic effects on the germination and growth of *Salvia miltiorrhiza* seeds.

Similar to other crops, continuous cropping of *C. pilosula* is linked to growth slowness, yield decrease, quality degradation, and pest and disease severity (Hosseinzadeh et al., 2018). The primary field manifestations of continuous cropping impediment in *C. pilosula* are dead seedlings, reduced seedling growth, occurrence of various pests and diseases, and incidence of root rot disease that can reach more than 30% (Wu et al., 2013; Yan et al., 2019). Because the root rot pathogen can spread through soil and seedlings, the prevalence of root rot in *C. pilosula* is on the rise (Zhang and Wang, 2019). Although there is a preliminary understanding of the causes of *C. pilosula* continuous cropping obstacle, its abatement is still in the exploratory stage (Guo et al., 2012; Ren et al., 2022) due to the complexity of the causes and mechanisms.

Numerous preliminary studies addressed the effectiveness of organic fertilizers in mitigating the challenges posed by continuous cropping in various crops. For example, Zhou et al. (2021) and Li et al. (2019) demonstrated that both organic fertilizer and silica-calcium-potassium-magnesium fertilizer can mitigate the continuous cropping obstacle of *C. tangshen* by enhancing photosynthetic metabolism and regulating the soil biochemical environment promoting higher yields. Biochar is a carbon-rich material produced through the thermal decomposition of biomass under conditions of limited oxygen availability. When mixed into agricultural soils, biochar can be used as a fertilizer to promote soil fertility and plant growth by providing and retaining nutrients in agroecosystems (Lin et al., 2023; Liu et al., 2024). Jin Wook Kim (Kim et al., 2022) applied bacterial fertilizer treatment to cucumber and the results showed that cucumber applied with bacterial fertilizer was better than the control in plant height, stem thickness, number of leaves and yield, indicating that bacterial fertilizer was able to stimulate plant growth. Vermicompost also contains many bacteria, actinomycetes, as well as a large number of amino acids, enzymes and bio-hormones and other biologically active substances, which can accelerate the transformation of nutrients and facilitate plant growth (Zhao et al., 2017). Additionally, the use of microbial fertilizer has been shown to enhance the quality of *C. tangshen*. However, limited research has been conducted on the issue of continuous cropping in *C. pilosula*. We aimed to investigate the effects of environmentally friendly biochar, bacterial fertilizer, and vermicompost as soil amendments on seed germination and seedling physiology of *C. pilosula* under *C. pilosula* continuous cropping obstacles. Additionally, we examined the impact of these three fertilizers on the growth of continuously cropped *C. pilosula* in the field. Assuming that these three fertilizers have some degree of mitigating effect on continuous *C. pilosula*, the study will provide new insights into pollution-free production of *C. pilosula* and sustainable soil utilization (Hosseinzadeh et al., 2016; Zhao et al., 2017; Liu et al., 2020).

## 2 Materials and methods

### 2.1 Test materials

The seeds and seedlings of *C. pilosula* were obtained from Lichuan Town Liuhe Herbal Cooperative in Tanchang County, Gansu Province, China. The seeds of *C. pilosula* had an average thousand grain weight of (9.70 ± 0.35) g, a germination rate of more than 96%, seedlings were annual, uniform in size, free from diseases, pests and mechanical damage, with an average root length of (14.53 ± 0.56) cm, and a root thickness of (3.62 ± 0.84) mm.

We used Mumeituli bio-bacterial fertilizer (ETS (Tianjin) Biotechnology Development Co., Ltd, with the effective live bacteria number of 200 million/g, total N, P, K content of 6.31%, and organic matter content of 20%. There are a variety of beneficial antibiotic strains such as *Bacillus megaterium*, *Bacillus glabrata*, *Bacillus amyloliquefaciens*, *Bacillus licheniformis*, *Mucor xylosus*, *Botrytis cinerea* and other beneficial antimicrobial strains, which are concentrated and dried using a unique bio-fermentation technology.), biochar-based fertilizer (Lize Environmental Technology Co., Ltd., with 53.4% organic carbon, 10.4% total nitrogen, 3.9% effective phosphorus, and 15.3% effective potassium); and vermicompost (Drunken Flower Vermicompost Organic Fertilizer Co., Ltd., with 6% N, P, K content, 40% organic matter, 200,000/g ~ 200 million/g beneficial bacteria, and 18 kinds of amino acids).

### 2.2 Study area

The study area is located in Lasha Village, Lichuan Town, Tanchang County. It is an alpine and humid area with an altitude of 2390 m, annual rainfall of 450 mm, annual average temperature of 10°C, annual solar radiation of 124.9 kcal/cm, frost-free period of 180 d, and annual sunshine hours of 2081.1 h. The climate is alpine and arid, with plenty of sunshine, and plenty of rain in the spring and autumn. Soil has a pH of 8.3, organic matter content of 12.6 g/kg, fast-acting phosphorus of 23.7 mg/kg, nitrate nitrogen of 16.2 mg/kg, ammonium nitrogen of 12.8 mg/kg.

### 2.3 Experimental design

#### 2.3.1 Effects of three fertilizer extracts on seed germination and seedling resistance physiology of *C. pilosula* in continuous cropping

The experiment was established as a two-factor randomized block trial design, with two factors of fertilizer type and concentration. Fertilizers included biochar, bacterial fertilizer, and vermicompost; the concentrations were: undiluted, diluted 10 times, diluted 50 times, and distilled water treatment as the control (**Table 1**).

**Table 1:** Experimental treatments.

Treatments	No dilution	10 times dilution	50 times dilution
Bacterial fertilizer	J0	J10	J50
Biochar	T0	T10	T50
vermicompost	Q0	Q10	Q50

#### 2.3.2 Effects of three fertilizers on the resistance physiology of transplanted *C. pilosula*

A two-factor randomized group experimental design was used with two factors: cultivation method and fertilizer application. The cultivation method included rotational stubble (R) and continuous stubble (C), and fertilizer application included Mumeituli bio-bacterial fertilizer (J), biochar-based fertilizer (T), vermicompost (Q), and control (CK), with 8 treatments in total and each treatment replicated three times. All treatments were CCK, CJ, CT, CQ, RCK, RJ, RT, RQ. The experimental site was stubble cultivated in the previous year, with continuous stubble being land planted with *C. pilosula* for 2 consecutive years, and rotational stubble with *Astragali radix*, as the previous crop. The plot area was 5 m×3.5 m=17.5 m^2^, with a plot spacing of 40 cm and a non-treated buffer around the experimental area, and the experimental plot was cultivated with stubble in the previous year.

*C. pilosula* was transplanted on April 12. Bio-bacterial fertilizer is used as Mumei Tuli compound microbial fertilizer (application rate is 364.2 kg/acre); biochar-based fertilizer (application rate is 607 kg/acre); vermicompost is used as “Taiping No. 2 Aichi”, which is sieved from cow dung after feeding for 1 month (application rate is 607 kg/acre). All treatments were applied to the soil as a one-time base fertilizer at the beginning of the planting period, and no additional fertilizer was applied during the trial period. When the soil in the field was dry, we needed to water in time and removed weeds diligently to prevent grass shortage, reflecting regular field management.

### 2.4 Measurement indexes and methods

#### 2.4.1 Soil extract preparation

Preparation of the rhizosphere soil extract (Lin et al., 2022): air-dried rhizosphere soil was sieved through a 20-mesh sieve, and 100 g was weighed into a 500 mL beaker, distilled water was added to the total volume of 400 mL, and solution was sonicated for 1 h and left overnight; then, the extract was filtered through a filter paper.

#### 2.4.2 Fertilizer extract preparation

Preparation of biochar extract (Guo et al., 2020): 10 g of biochar sample was weighed and mixed with distilled water in a 100 mL conical flask with a charcoal-to-water ratio of 1:5, shaken at 150 r/min for 2 h at room temperature, vacuum filtered with a double layer of medium speed filter paper; the charcoal residue was rinsed with 20 mL of deionized water, and the filtrate was stock solution (T0). The filtrate was diluted 10 (T10) and 50 times (T50) when used.

Preparation of the Mumeituli bio-bacterial fertilizer extract (Li et al., 2018): 10 g of bacterial fertilizer compound was weighed in a 250 mL triangular flask, 100 mL of sterile water was added, and the flask was shaken for 30 min at room temperature at 200 r/min; the solution was filtered through a filter paper to obtain the required stock solution (J0). The extract was diluted 10 (J10) and 50 times (J50) for use.

Preparation of vermicompost extract (Liu et al., 2017): the vermicompost was fully air-dried and crushed, passed through a 20-mesh sieve, and a 20 g sample was weighed into a 250 mL conical flask with 100 mL of deionized water, shaken for 30 min, and then placed at room temperature for 24 h. The larger sized residue was first filtered through gauze, and then the filtrate was filtered through a double layer of medium-speed filter paper until it was free of particles to obtain the desired vermicompost extract (Q0). The extract was diluted 10 (Q10) and 50 times (Q50) for use.

#### 2.4.3 Seed germination test

Seed germination test (Mackenzie and Naeth, 2019): full, unbroken seeds of *C. pilosula* were selected. Seeds were soaked in distilled water for 6 h before the test, then surface-sterilized with 5% sodium hypochlorite solution for 15 min, and then rinsed several times with sterile water. Petri dishes were lined with a double layer of filter paper (both petri dishes and filter paper were autoclaved) and the treated seeds were evenly arranged in 90 mm size petri dishes with 50 seeds per dish. Three different concentrations of extracts (no dilution, 10-fold dilution and 50-fold dilution) were used in germination experiments, with three replications of each treatment; distilled water was used as the control (CK). In each petri dish, 2 mL of root soil extract and 2 mL of different dilutions of the treatment solution were added to each dish, and the seeds were incubated at 25°C for 15 d. During the incubation period, the petri dishes were supplemented with 2 mL of distilled water every 2 d. The seeds were observed and any changes recorded daily. The germination rate, germination potential, and germination index of the seeds were calculated based on total number of germinated seeds and the number of seeds sprouted on the day of maximum sprouting. The formula is as follows (Equations 1–3):

#### 2.4.4 Measurement indexes and methods

1) Seed germination indicators:


(1) Germination rate=(total number of germinated seeds(15d)÷total number of seeds for testing)×100%

(2) Germination potential=(the number of seeds sprouted on the day of maximum sprouting÷total number of seeds for testing)×100% 

(3) Germination index (GI)=∑ (Gt / Dt),Gt is the number of germinated seeds on day t and Dt is the corresponding number of days to germination)

After 15 days of seed germination, SOD enzyme activity, POD enzyme activity, MDA content, and total chlorophyll content of whole seeding of *C. pilosula* were measured in random sampling. Superoxide dismutase (SOD) activity was determined with the nitrogen blue tetrazolium method, peroxidase (POD) activity with the guaiacol method, malondialdehyde (MDA) content with the thiobarbituric acid TBA colorimetric method, and total chlorophyll content was determined using the acetone method (Zhang et al., 2019).

2) Physiological and biochemical indices:

Inside the experimental field, 20 C*. pilosula* whole seedlings were randomly selected using the five-point sampling method and their physiological parameters were measured on July 10, August 10, September 10, and October 10. The whole seedlings were weighed on a balance, placed in a mortar, a small amount of calcium carbonate and quartz sand were added, and an appropriate amount of 80% acetone solution was added and ground thoroughly to form a homogenous paste, which was then subjected to subsequent operations. Superoxide dismutase (SOD) activity was determined with the nitrogen blue tetrazolium method, peroxidase (POD) activity with the guaiacol method, free proline content with the acid ninhydrin colorimetric method, soluble protein content with the Komas Brilliant Blue G-250 reagent staining method, and malondialdehyde (MDA) content with the thiobarbituric acid TBA colorimetric method (Zhang et al., 2019).

### 2.5 Statistical analysis

Data analysis of the trial was performed using Office 2016 and IBM SPSS Statistics version 26.0 (IBM SPSS Inc., Chicago, USA) statistical analysis software. One-way analysis of variance (ANOVA) using SPSS 27.0 with LSD method of significance test (p<0.05).

## 3 Results

### 3.1 Effects of three fertilizer extracts on seed germination and seedling resistance physiology in *C. pilosula* under continuous cropping

#### 3.1.1 Seed germination

All three fertilizer extracts significantly promoted the germination of *C. pilosula* seeds. Among them, both bacterial fertilizer and vermicompost at the 10-dilution concentration had the most significant effect on promoting germination. Germination potential increased by 30.6 and 47.9%, respectively, for bacterial fertilizer and vermicompost compared to the control group. Biochar at a 50-fold dilution also had a significant effect on germination; increasing germination rate by 46.5% compared to the control group. All treatments, except for the undiluted biochar, showed significant differences in germination rate and potential compared to the control group. The germination rate and potential of *C. pilosula* seeds treated with T0 (control) were not significantly different from the control group. However, the increase in germination rate and potential in T10 was 14.3 and 12.7%, respectively, and in T50, it was 23.5, and 31.0% compared to the control group. The germination indices of *C. pilosula* seeds in all three treatments were higher than those of the control group. The most significant increase in germination rate and germination index of *C. pilosula* seeds was observed in J10 and Q10, with increases of 24.5 and 26.5% for J10, and 31.0 and 47.9% for Q10, respectively over those in CK. Thus, all three fertilizer extracts with different concentrations can alleviate the effects of continuous cropping stress on the germination of *C. pilosula* seeds (**Table 2**).

**Table 2:** Effects of three extracts on seed germination in *C. pilosula*.

Treatments	Germination rate	Germination potential	Germination index
CK	65.33 ± 1.15e	47.33 ± 2.00d	50.87 ± 2.89d
T0	68.67 ± 2.31e	53.33 ± 4.16cd	60.36 ± 5.13abc
T10	74.67 ± 2.31d	62.00 ± 7.21abc	66.41 ± 7.53ab
T50	80.67 ± 1.18abc	69.33 ± 6.43a	68.77 ± 4.82a
J0	77.33 ± 2.15bcd	60.67 ± 4.62abc	56.92 ± 4.19cd
J10	81.33 ± 1.32ab	62.00 ± 8.00abc	62.45 ± 3.97abc
J50	74.00 ± 5.29d	64.00 ± 5.03ab	61.24 ± 6.55abc
Q0	76.67 ± 3.06cd	64.00 ± 4.00ab	62.60 ± 2.98abc
Q10	82.67 ± 5.03a	70.00 ± 2.00a	66.91 ± 2.55ab
Q50	79.33 ± 4.03abc	56.67 ± 7.57bc	57.68 ± 8.37bcd

different lowercase letters within columns indicate significant differences among treatments at the 5% level (P < 0.05).

#### 3.1.2 Resistance physiology in *C. pilosula* seedlings

##### 3.1.2.1 SOD enzyme activity

**Figure 1:**
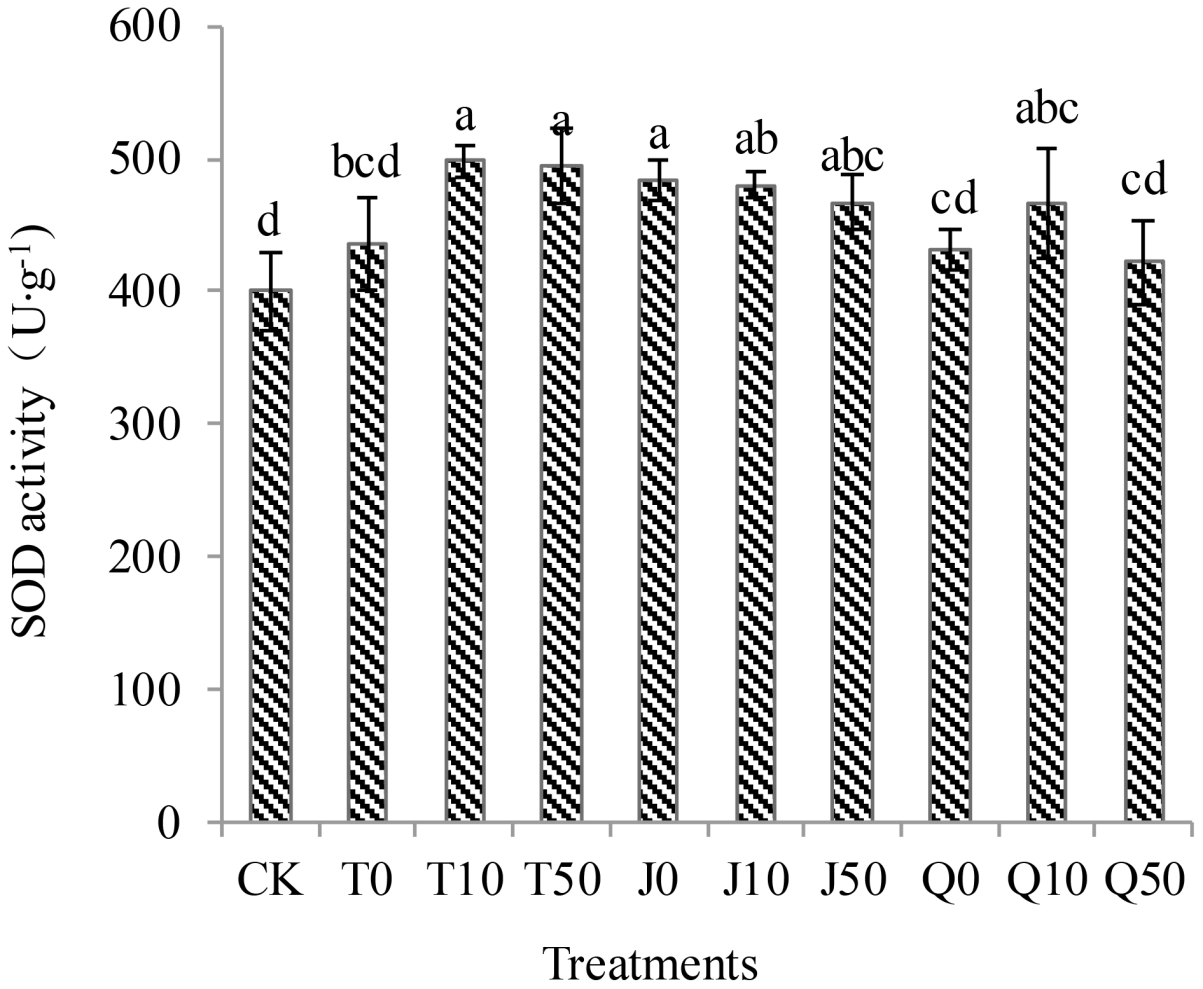
Effects of three extracts on SOD enzyme activity in *C. pilosula* seedlings.

The differences in SOD enzyme activities among the T0, Q0, and Q50 treatments, and CK were not statistically significant; however, SOD enzyme activities of the remaining treatments were significantly higher than those of the CK seedlings (**Figure 1**). Among them, the T10, T50, and J0 treatments exhibited the most significant increases of 24.6, 23.6, and 21.0%, respectively. The SOD enzyme activity among the three concentrations in the J treatment were significantly higher than in the CK. The SOD enzyme activity in seedlings decreased with increasing dilution concentration of the fertilizer extracts. The T and J treatments were more effective than the Q treatment in enhancing the SOD enzyme activity in *C. pilosula* seedlings. Additionally, the SOD enzyme activity in all treatments was higher than that in the CK group. These findings indicate that all three fertilizer extracts, at different concentrations, have the potential to enhance the antioxidant capacity in *C. pilosula* seedlings.

##### 3.1.2.2 POD enzyme activity

**Figure 2:**
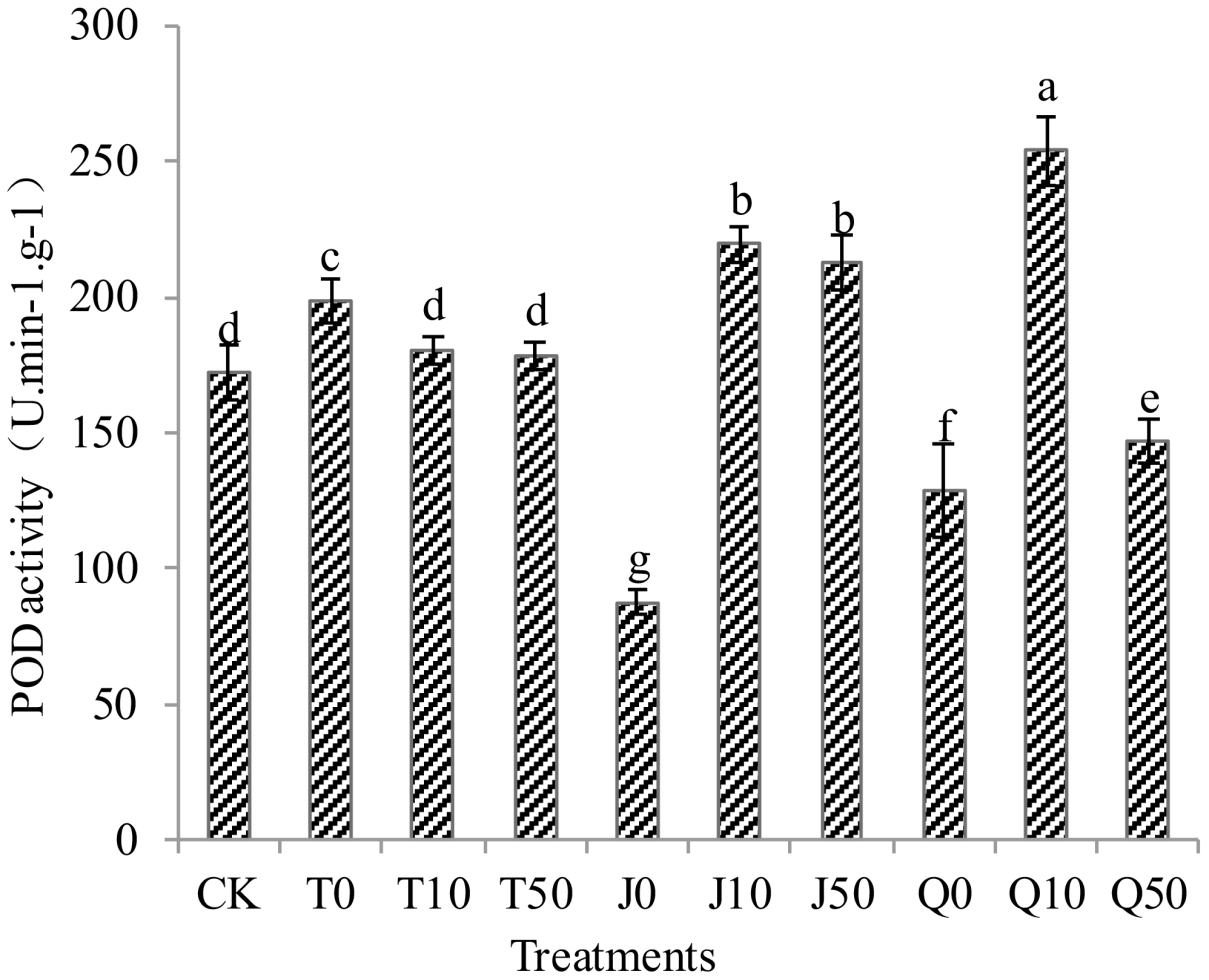
Effects of three extracts on POD enzyme activity in *C. pilosula* seedlings. Lower case letters indicate significant differences among treatments at the *P*<0.05 level.

All treatments showed significant differences in POD compared to the CK group, except for the T10 and T50 treatments, which were not significantly different from the CK group. Specifically, the T0, J10, J50, and Q10 treatments exhibited significant increases in POD enzyme activity of 15.5, 27.5, 23.3, and 47.7%, respectively. On the other hand, the J0, Q0, and Q50 treatments led to significant decreases in POD enzyme activity of 49.2, 25.2, and 14.7%, respectively. Notably, the effect of J treatment at concentrations J10 and J50 showed the highest efficacy in enhancing POD enzyme activity in *C. pilosula* seedlings. Among the Q-treated seedlings, the Q10 treatment demonstrated the most significant increase in POD enzyme activity compared to Q0 and Q50, surpassing the effectiveness of other treatments (**Figure 2**). These results indicate that all treatments, except for J0, Q0, and Q50, increased the POD enzyme activity, thus enhancing the antioxidant capacity of *C. pilosula* seedlings.

##### 3.1.2.3 MDA content

**Figure 3:**
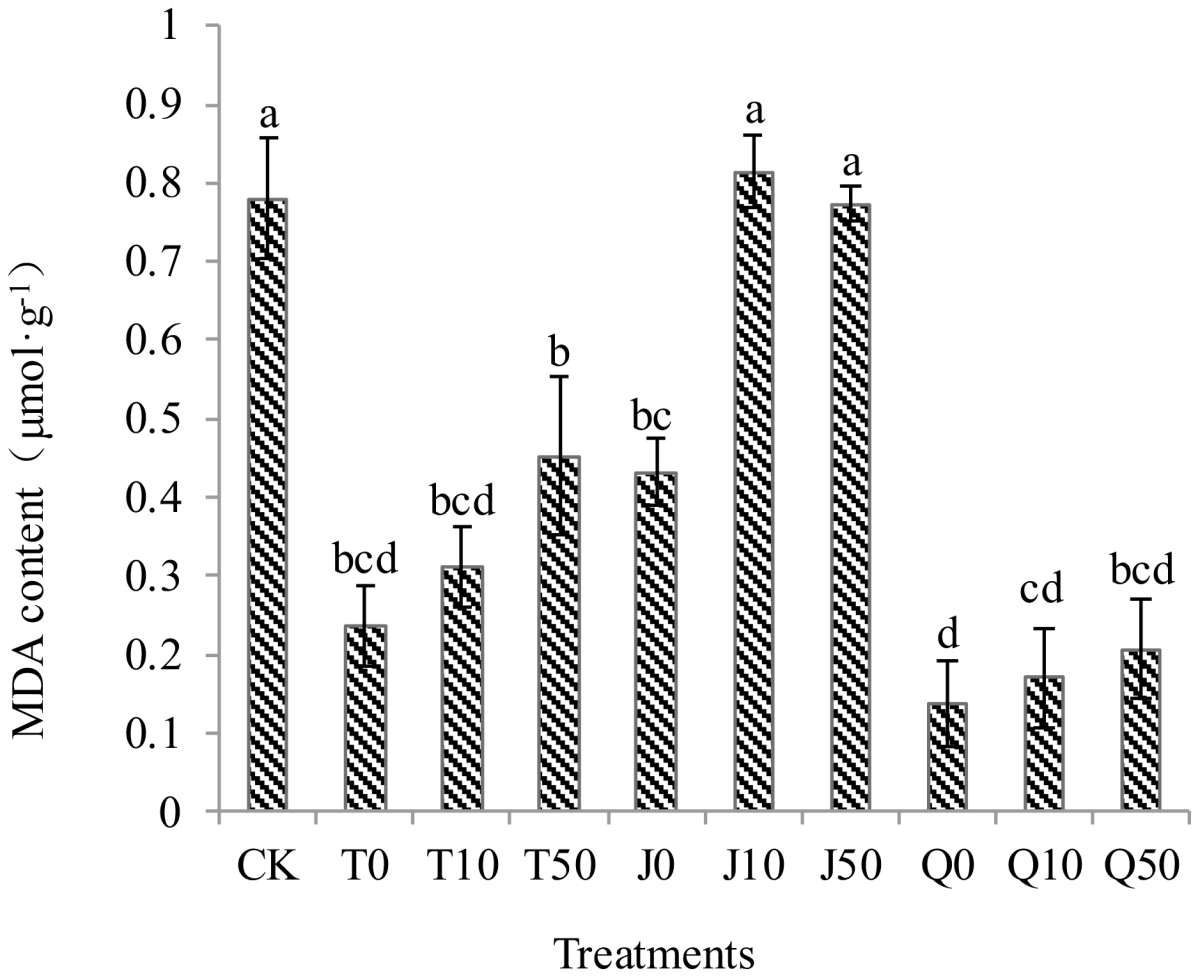
Effects of three extracts on MDA content in *C. pilosula* seedlings.

There was no significant difference observed between the J10 and J50 treatments compared to the CK group. Among all the treatments, the J10 treatment had the highest MDA content. Further, the MDA content in *C. pilosula* seedlings increased in T and Q treatments with increasing dilution of the solution. However, it was significantly lower than the MDA content of the CK group. Specifically, the reductions in MDA content in the T0, T10, T50, Q0, Q10, and Q50 treatments were 69.5, 60.0, 42.0, 82.5, 78.1, and 73.6%, respectively. Overall, all treatments, except for J10 and J50, effectively reduced the excessive accumulation of MDA in *C. pilosula* seedlings under continuous cropping. Among them, the three treatments corresponding to vermicompost exhibited the most significant effects (**Figure 3**).

##### 3.1.2.4 Total chlorophyll content

**Figure 4:**
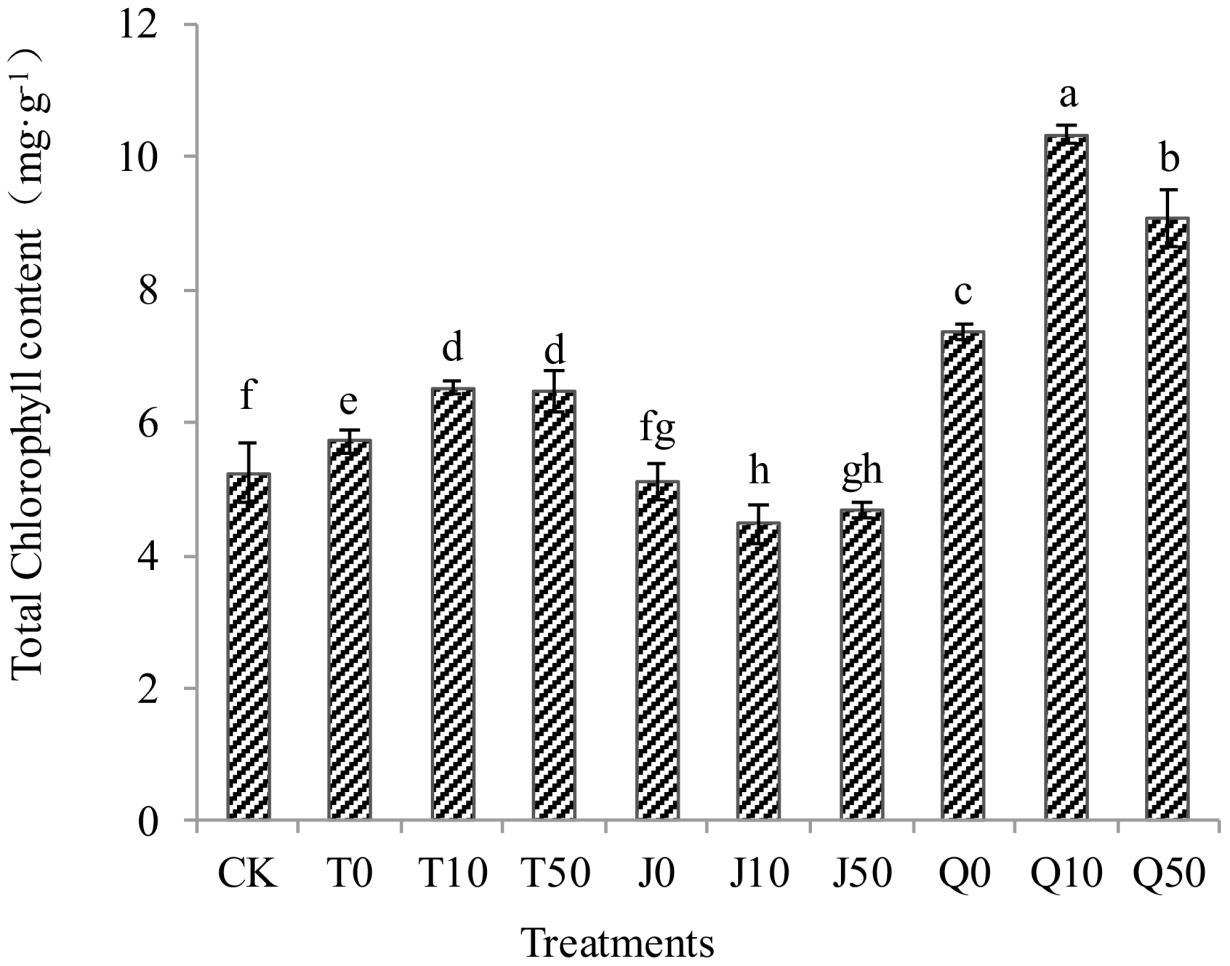
Effects of three extracts on total chlorophyll content in *C. pilosula* seedlings.

All treatments exhibited a significant difference in chlorophyll content, except for the J0 treatment. The total chlorophyll content in *C. pilosula* seedlings in both T and Q treatments was significantly higher than in the CK group. The J0 treatment showed a significantly higher chlorophyll content compared to the CK group, while the J10 and J50 treatments exhibited significant reductions of 14.6 and 10.6%, respectively, compared to the CK group. Total chlorophyll content in the Q treatment was significantly higher than that in the T, J, and CK treatments. Both T and Q treatments exhibited higher chlorophyll content at a 10-fold dilution. Among all the treatments, Q enhanced the total chlorophyll content most, followed by T. Total chlorophyll content in *C. pilosula* seedlings in J treatment was consistently lower than in the CK group (**Figure 4**).

### 3.2 Physiological indexes of transplanted *C. pilosula*

#### 3.2.1 SOD enzyme activity

SOD enzyme activity in *C. pilosula* seedlings generally showed an increasing trend in different treatments between July to August, with a slight decrease in the CCK (continuous cropping CK) and RT (rotational cropping CK) treatments. Subsequently, there was a moderate increase in the CCK, CJ (continuous cropping J), and RT treatments between August to September, while the other treatments exhibited a decrease. During the harvest period from September to October, the SOD enzyme activity in *C. pilosula* seedlings showed a decreasing trend in all treatments. However, the SOD enzyme activity in each treatment of rotational cropping was still higher than the corresponding treatments in continuous cropping. The RT, RJ, and RQ treatments exhibited significant differences not only compared to RCK (rotational cropping CK), but also compared to CCK (continuous cropping CK). All three treatments, including bacterial fertilizer, biochar, and vermicompost, improved the SOD enzyme activity in field-grown *C. pilosula* plants. Because an enhancement in SOD enzyme activity helps to remove and reduce the damage from the reactive oxygen species in plants, these treatments contributed to improving the growth potential of continuously cropped *C. pilosula* plants (**Table 3**).

**Table 3:** Effects on SOD activity in *C. pilosula*.

Treatments	SOD enzyme activity (U/g FW)			
	July 10th	August 10th	September 10th	October 10th
CCK	229.13 ± 25.47bc	227.83 ± 36.08cd	234.30 ± 18.35d	212.30 ± 13.64c
CT	262.78 ± 17.51ab	297.73 ± 12.48ab	289.97 ± 9.93b	240.78 ± 7.77b
CJ	169.58 ± 22.08c	201.94 ± 11.65d	271.84 ± 30.33bc	213.59 ± 6.73c
CQ	273.14 ± 15.69ab	295.15 ± 30.82ab	257.61 ± 8.97cd	208.41 ± 5.93c
RCK	257.61 ± 46.98ab	260.19 ± 19.42bc	243.37 ± 14.70d	216.18 ± 14.70c
RT	297.73 ± 60.95a	295.15 ± 25.47ab	324.92 ± 5.93a	278.32 ± 12.24a
RJ	269.26 ± 15.69ab	305.50 ± 15.69a	299.03 ± 13.88b	236.89 ± 10.27b
RQ	301.62 ± 32.95a	323.62 ± 22.24a	277.02 ± 11.86bc	269.26 ± 16.17a

Lower case letters within columns indicate significant differences among treatments at the P < 0.05 level.

#### 3.2.2 POD enzyme activity

The overall trend in POD enzyme activity in *C. pilosula* leaves showed an initial increase followed by a decrease. The most significant changes in enzyme activities were observed in August and September, with enzyme activities in September being significantly higher than those in August for most treatments, except for the RT (rotational cropping CK) treatment. Additionally, in August, the POD enzyme activities were significantly higher compared to July for all treatments, except for the RQ (rotational cropping Q) treatment. The POD enzyme activities under rotational cropping treatments were higher than those under continuous cropping for all stages. In October, when the above-ground parts of the plants started to wilt, the POD enzyme activities in *C. pilosula* leaves for all treatments slightly decreased compared to September. However, POD enzyme activities in the rotational cropping treatments remained higher than those in the continuous cropping treatments. There was no significant difference in enzyme activities between the same fertilizer treatments applied to different cultivation methods (**Table 4**).

**Table 4:** Effects of three kinds of fertilizers on POD activity in *C. pilosula*.

Treatments	POD enzyme activity (U/g FW)			
	July 10th	August 10th	September 10th	October 10th
CCK	110.00 ± 12.70d	181.33 ± 11.15e	236.00 ± 23.46c	222.00 ± 51.03d
CT	236.33 ± 15.14c	290.00 ± 21.00c	361.33 ± 28.73b	364.00 ± 34.18b
CJ	122.67 ± 4.62d	205.33 ± 12.31de	391.33 ± 16.97b	343.33 ± 14.19bc
CQ	109.33 ± 43.88d	338.67 ± 9.24b	390.67 ± 9.45b	383.33 ± 11.02ab
RCK	143.33 ± 9.17d	245.33 ± 15.08cd	278.00 ± 17.44c	280.67 ± 15.77cd
RT	334.67 ± 16.03b	469.33 ± 8.08a	402.00 ± 11.14b	396.67 ± 20.28ab
RJ	246.67 ± 25.32c	426.67 ± 16.29a	506.67 ± 19.87a	428.00 ± 12.49ab
RQ	378.67 ± 11.02a	378.00 ± 12.00b	568.00 ± 18.00a	456.00 ± 12.00a

Lower case letters within columns indicate significant differences among treatments at the P < 0.05 level.

#### 3.2.3 MDA content

The effects of T, J, and Q treatments on the MDA content in *C. pilosula* leaves varied and changed over time. In July, the MDA content of *C. pilosula* leaves in all treatments, except for RCK (continuous cropping CK) and RJ (rotational cropping J), was higher in continuous cropping compared to rotational cropping. In August, the MDA content showed a slight decrease in the RCK and RJ treatments compared to July, while it increased in the remaining treatments. The MDA content in plants in all continuous cropping treatments was higher than that in rotational cropping. RCK, RT, RJ, and RQ treatments, MDA was significantly reduced by 31.8, 28.5, 41.6, and 31.8% respectively, compared to the CCK, CT, CJ, and CQ treatments. The MDA content in *C. pilosula* reached its peak in September, with the highest value of 4.45 μmol/g in the CCK treatment, which was significantly different from the other treatments in both cultivation methods, and it was 2.28 times higher than in the RT treatment. In October, the MDA content decreased for all treatments, except for the RT treatment, which showed a slight increase (**Table 5**). Considering four months of realizations, all three fertilizers effectively prevented the excessive accumulation of MDA content in *C. pilosula*. Specifically, the RT and RJ treatments appeared more effective and consistent in their potential capacity to reduce lipid peroxidation in plant cell membranes and mitigate damage to the membranes.

**Table 5:** Effects of three fertilizers on MDA content of *C. pilosula*.

Treatments	MDA content (*μmol/g*)			
	July 10th	August 10th	September 10th	October 10th
CCK	1.45 ± 0.31ab	2.14 ± 0.58ab	4.45 ± 0.08a	3.33 ± 0.44a
CT	1.89 ± 0.45a	2.49 ± 0.49a	3.42 ± 0.10c	2.47 ± 0.03bc
CJ	1.25 ± 0.02b	2.40 ± 0.19a	4.00 ± 0.07b	2.35 ± 0.02bc
CQ	1.32 ± 0.33b	1.77 ± 0.06c	3.28 ± 0.03c	1.77 ± 0.15de
RCK	1.48 ± 0.15ab	1.46 ± 0.07c	2.29 ± 0.05d	2.80 ± 0.36b
RT	1.04 ± 0.14b	1.53 ± 0.03c	1.95 ± 0.11f	2.14 ± 0.27cd
RJ	1.37 ± 0.27ab	1.25 ± 0.28c	2.12 ± 0.15e	1.54 ± 0.24e
RQ	1.14 ± 0.36b	1.46 ± 0.10bc	2.18 ± 0.05de	1.65 ± 0.09e

Lower case letters within columns indicate significant differences among treatments at the P < 0.05 level.

#### 3.2.4 Free proline content

The free proline content in *C. pilosula* plants showed an increasing trend in July to October, with significant changes mostly observed in September and October. The free proline content in rotational cropping treatments was higher than that in continuous cropping. In July, proline contents in continuous cropping CJ were greater than in CCK, while those in CT and CQ treatments were significantly lower than in CCK, with reductions of 25.3 and 36.2%, respectively. The RJ (rotational cropping J) and RQ (rotational cropping Q) treatments exhibited significant increases of 21.2 and 46.7%, respectively, in free proline content compared to the CCK treatment. In October, the free proline content in *C. pilosula* was significantly greater in T under both cultivation methods than in CCK but not RCK (rotational cropping CK). The continuous cropping CQ treatment also showed a significant difference in proline from CCK. The rotational cropping treatments of RT, RJ, and RQ exhibited significantly higher free proline contents than RCK, with increases of 39.0, 23.4, and 32.1%, respectively. All three fertilizers enhanced free proline content in *C. pilosula* to different extents, with J (vermicompost) and Q (biochar) showing more pronounced and stable enhancements under both cultivation methods, while T (bacterial fertilizer) performed better in later stages of growth (**Table 6**).

**Table 6:** Effects of three fertilizers on proline content in *C. pilosula.*.

Treatments	Proline content (μg/g)			
	July 10th	August 10th	September 10th	October 10th
CCK	36.13 ± 1.39c	43.91 ± 4.10c	49.32 ± 2.40d	61.17 ± 2.81e
CT	26.98 ± 2.43d	47.14 ± 1.74c	50.50 ± 7.21d	74.35 ± 0.82c
CJ	41.25 ± 2.61bc	49.57 ± 0.86bc	52.49 ± 1.19d	65.86 ± 1.38de
CQ	23.04 ± 2.68d	48.22 ± 2.23bc	54.73 ± 10.01cd	72.81 ± 1.80c
RCK	40.13 ± 3.96bc	45.11 ± 5.89c	58.13 ± 5.71cd	70.90 ± 3.80cd
RT	28.02 ± 0.67d	56.77 ± 4.28ab	64.70 ± 7.21bc	98.57 ± 2.57a
RJ	43.77 ± 0.75b	63.40 ± 0.37a	88.84 ± 6.04a	87.50 ± 4.71b
RQ	53.00 ± 9.51a	62.97 ± 10.31a	70.39 ± 2.44b	93.64 ± 4.92a

Lower case letters within columns indicate significant differences among treatments at the P < 0.05 lever.

#### 3.2.5 Soluble protein content

The soluble protein content in *C. pilosula* was consistently lower in the continuous cropping treatment compared to the rotational cropping treatment over the four-month period of study. In the continuous cropping treatment, the soluble protein content increased until September, after which it started to decrease. In contrast, the rotational cropping treatment showed an increasing trend in soluble protein content until August, followed by a slow decrease, although the decrease was still greater than that observed in the continuous cropping treatment. In October, the soluble protein content in both cultivation methods decreased compared to September. There were no significant differences among the three fertilizers in either of the cropping methods. However, all three fertilizers showed a significant difference compared to CCK and RCK (**Table 7**). It is evident that all three fertilizers were capable of enhancing the soluble protein content in *C. pilosula* to varying extents, thereby mitigating the damage caused by osmotic and nutrient dysregulation in *C. pilosula* under continuous cropping stress.

**Table 7:** Effects of three fertilizers on soluble protein content in *C. pilosula*.

Treatments	Soluble protein content (mg/g)			
	July 10th	August 10th	September 10th	October 10th
CCK	5.91 ± 0.12d	6.03 ± 0.25d	7.13 ± 0.49e	7.55 ± 0.15c
CT	6.93 ± 0.17c	7.40 ± 1.39cd	9.60 ± 0.33d	8.44 ± 0.79b
CJ	7.42 ± 0.25bc	8.21 ± 0.39cd	10.36 ± 0.07bc	8.50 ± 0.11b
CQ	7.87 ± 0.47b	8.92 ± 0.28bc	9.95 ± 0.25cd	8.56 ± 0.53b
RCK	7.32 ± 0.31bc	10.89 ± 1.53ab	9.84 ± 0.11cd	7.85 ± 0.13c
RT	8.07 ± 0.62b	11.37 ± 0.31a	10.67 ± 0.12b	8.99 ± 0.46ab
RJ	9.73 ± 0.65a	11.91 ± 0.43a	10.84 ± 0.11b	9.00 ± 0.34ab
RQ	9.88 ± 0.76a	12.45 ± 0.62a	11.38 ± 0.51a	9.49 ± 0.49a

Lower case letters within columns indicate significant differences among treatments at the P < 0.05 lever.

## 4 Discussion and conclusions

Allelochemical, which are one of the causes of cropping obstacle, are usually distributed in the nutrient organs and some reproductive organs of plants and can be released into the environment through a variety of pathways, including emission through root secretions (Sun and He, 2019; Miao et al., 2021). Autotoxins contained in the inter-root soil leachate of plants with a high incidence of cropping obstacle can indeed impede the germination of seeds and the growth of seedlings of their own or other plants (Deng et al., 2017). The aqueous phase extract of biochar has a fraction of highly active substances that are thermally stable and able to remain stable in aqueous solution for a long period of time, and this fraction is the main component that promotes seed germination (Gao et al., 2023). Mumeituli bio-bacterial fertilizer and vermicompost contains a large number of beneficial active flora, microbial activity secretion of certain substances can also play a role in promoting the effect of *C. pilosula* seed germination and seedling growth, in addition to vermicompost contains a variety of amino acids and growth hormones, which are able to alleviate the successive cropping of the soil extracts of liquid stress on the inhibition of the germination of *C. pilosula* seeds and seedling growth.

The germination of *C. pilosula* seeds under continuous cropping was improved by biochar, bacterial fertilizer, and vermicompost at different concentrations. Among these, the 10 times diluted extract of biofertilizer stimulated seed germination most of the extracts tested and the other two concentrations. Additionally, because biochar is only weakly alkaline, adding too much would result in a high pH of the environment for seed germination, which is harmful to seed germination (Liao et al., 2014; Kolton et al., 2017; Tian et al., 2017). Except for individual treatments, all three fertilizers had a positive effect on the increase in antioxidant enzyme activity, osmoregulatory compounds, and total chlorophyll content in continuous cropping of *C. pilosula* seedlings under simulated stress in root soil extract. In comparison to the other treatments, bacterial fertilizer diluted 10 and 50 times, and vermicompost diluted 10 times significantly boosted POD enzyme activity in *C. pilosula* seedlings. Biochar and vermicompost treatments were found to be more effective than biofertilizer treatments in reducing excessive accumulation of MDA and increasing the chlorophyll content in *C. pilosula* seedlings. The chlorophyll content in all *C. pilosula* seedlings treated with biofertilizer was lower compared to the control group. This suggests that the biofertilizer treatment negatively affected the photosynthetic capacity of *C. pilosula*. Reduced chlorophyll levels are often linked to inhibited plant growth and decreased grain yield (Han et al., 2013; Du et al., 2021).

Continuous cropping obstacles will lead to a decline in plant growth, the plant is subjected to adversity stress, the level of antioxidant enzyme activity in the plant, the accumulation of osmoregulatory substances and the good or bad resistance of the plant have a direct impact. The three fertilizers had a better regulating effect on antioxidant enzyme activity, the accumulation of malondialdehyde content and the content of osmoregulatory substances in different stubble *C. pilosula* compared with the control, because the improvement of soil physicochemical properties by the three fertilizers facilitated the nutrient uptake by *C. pilosula* plants, stimulated the growth of *C. pilosula*, improved *C. pilosula* resistance, and helped *C. pilosula* to resist the stress of the continuous cropping obstacles, which is in line with the studies by Zhu Yihao (Zhu et al., 2017) and Li Zhen et al. (Li et al., 2019). The results of Zhu Yihao’s study showed that the application of biochar could improve the antioxidant enzyme activity of lily, increase the accumulation of osmoregulatory substances in lily, and improve the resistance of lily. Li Zhen et al. showed that the addition of biochar was able to improve the physiological indices of continuously cropped *Andrographis paniculata*, bringing them close to those of the positive crop and shifting them in the direction of favoring plant growth. Mumeituli bio-bacterial fertilizer has been applied more in mitigating apple replanting diseases, and the application of Mumeituli bio-bacterial fertilizer has resulted in robust apple plants, developed root systems, and obvious promotion and recovery effects on disease-affected and weakened plants (Yang et al., 2020). Vermicompost (Feng et al., 2018) was found to be effective in improving the growth and physiology of continuous cucumber and continuous ginger (Yin et al., 2017), and was helpful in reducing the persecution of plant growth due to continuous cropping obstacles. Based on our findings, it remains a challenge to uncover the mechanism of abatement effects of different soil conditioner on continuous cropping of *C. pilosula*. A comprehensive investigation on the effects of continuous cropping on medicinal plants would be appropriate for a future study to fully understand the underlying intricacies.

In addition, other important indicators of *C. pilosula* succession, such as yield, aboveground biomass, disease index, and nutritional quality. Our group has completed the determination and the results are as follows: The extracts of three fertilizers with different concentrations had a certain inhibitory effect on two root rot pathogens (*Fusarium oxysporum* and *Fusarium acuminatum*) of *C. pilosula*. All of the three treatments can promote the growth of the embryonic roots of the *C. pilosula* seedlings inoculated with *Fusarium acuminatum* and the growth of hypocotyls of the *C. pilosula* seedlings inoculated with *Fusarium oxysporum*, and obviously improve the seed viability of the *C. pilosula* inoculated with two root rot pathogenic bacteria. All that three fertilizer can improve the activities of sucrase, catalase, urease and alkaline phosphatase in the rhizosphere soil of *C. pilosula* in different crop rotation; The contents of soil organic matter, available phosphorus, nitrate nitrogen and ammonium nitrogen were increased, and the diversity of soil microbial environment was improved. All the three fertilizers were beneficial to the yield increase and quality improvement of *C. pilosula* (Ma et al., 2024).

## Data availability statement

The original contributions presented in the study are included in the article/supplementary material. Further inquiries can be directed to the corresponding authors.

## Author contributions

ZY: Data curation, Writing – original draft. DQ: Funding acquisition, Writing – review & editing. KJ: Funding acquisition, Resources, Writing – review & editing. MD: Validation, Writing – review & editing, Supervision, Visualization. HL: Supervision, Validation, Visualization, Writing – review & editing.
